# Genetic studies on continuous flowering in woody plant *Osmanthus fragrans*

**DOI:** 10.3389/fpls.2022.1049479

**Published:** 2022-11-03

**Authors:** Qianqian Wang, Ge Gao, Xin Chen, Xiaohan Liu, Bin Dong, Yiguang Wang, Shiwei Zhong, Jinping Deng, Qiu Fang, Hongbo Zhao

**Affiliations:** Zhejiang Provincial Key Laboratory of Germplasm Innovation and Utilization for Garden Plants, School of Landscape and Architecture, Zhejiang A&F University, Hangzhou, Zhejiang, China

**Keywords:** flowering regulation, continuous flowering, transcription factors, transcriptome, *Osmanthus fragrans*

## Abstract

Continuous flowering is a key horticultural trait in ornamental plants, whereas the specific molecular regulation mechanism remains largely unknown. In sweet osmanthus (*Osmanthus fragrans* Lour.), plants based on their flowering characteristics are divided into once-flowering (OF) habit and continuous flowering (CF) habit. Here, we first described the flowering phenology shifts of OF and CF habits in sweet osmanthus through paraffin section and microscope assay. Phenotypic characterization showed that CF plants had constant new shoot growth, floral transition, and blooming for 1 year, which might lead to a continuous flowering trait. We performed the transcriptome sequencing of OF and CF sweet osmanthus and analyzed the transcriptional activity of flowering-related genes. Among the genes, three floral integrators, *OfFT*, *OfTFL1*, and *OfBFT*, had a differential expression during the floral transition process in OF and CF habits. The expression patterns of the three genes in 1 year were revealed. The results suggested that their accumulations corresponded to the new shoots occurring and the floral transition process. Function studies suggested that *OfFT* acted as a flowering activator, whereas *OfBFT* was a flowering inhibitor. Yeast one-hybrid assay indicated that *OfSPL8* was a common upstream transcription factor of *OfFT* and *OfBFT*, suggesting the vital role of *OfSPL8* in continuous flowering regulation. These results provide a novel insight into the molecular mechanism of continuous flowering.

## Introduction

Flowering is a vital developmental event and mediated by complex genetic mechanisms (Chen D. et al., 2018; Chen Z. et al., 2018). Among flowering plants, most species have a seasonal flowering period, blooming once a year in a certain season (once-flowering habit, OF), whereas some plants are able to flower continuously under a favorable season (continuous flowering habit, CF), such as rose, strawberry, and orchid (Ahmad et al., 2021). Obtaining plants with an extended flowering period is a goal of many gardeners, but the molecular regulators of continuous flowering are still not very clear (Iwata et al., 2012).

In OF plants, especially in model plant *Arabidopsis*, six major pathways are known to control flowering time: ambient temperature, photoperiod, gibberellin, vernalization, age, and autonomous pathways (Taylor et al., 2018). Signals from the six pathways converge into several important floral integrator genes, *FLOWERING LOCUS T* (*FT*), *TERMINAL FLOWER 1* (*TFL1*), *SUPPRESSOR OF OVEREXPRESSION OF CO1* (*SOC1*), *APETALA1* (*AP1*), and *LEAFY* (*LFY*) (Shannon and Meeks-Wagner, 1991; Han et al., 2018; Jung et al., 2021). *TFL1* and *FT* belong to the phosphatidylethanolamine-binding protein (PEBP) family and are key integrators of the floral transition pathways (Shannon et al., 1991). They have highly homologous sequences but act in an antagonistic manner in many species (Carmona et al., 2007; Jeon et al., 2011). *TFL1*, a flowering repressor, binds to FD to determine the floral transition by inhibiting the expression of *LFY* and *AP1*, which are downstream of the *FT* gene (Wang et al., 2021). As a downstream of *FT*, *SOC1* is a flowering activator and can upregulate floral meristem identity genes *AP1* and *LFY* to regulate flowering (Zhang et al., 2019).

Compared with OF plants, numerous studies on the molecular mechanism of continuous flowering have been conducted and several significant findings have been published. In rose, the CF habit is controlled by a monogenic recessive locus, and *TFL1* homologues are good candidate genes (Semeniuk, 1971; Hurst, 1941). By studying seven CF roses with distinct origins, a 9-kbp-long retrotransposon was discovered in the second intron of *RoKSN*, which might result in continuous flowering (Kumar and Bennetzen, 1999; Cairns, 2000). Epigenetic modifications and retrotransposon insertions are also thought to be concerned with the CF behavior of rose (Bai et al., 2021; Soufflet-Freslon et al., 2021). In strawberries, the continuous flowering habit occurs in both diploid strawberries (woodland strawberry) and octoploid strawberry (garden strawberry). According to Darrow (1966), a 2-bp deletion in *FvTFL1* might be a putative causal mutation for the perpetual flowering trait in woodland strawberry (Darrow, 1966; Koskela et al., 2012). In garden strawberries, a single dominant gene termed *Perpetual Flowering and Runnering* (*PFRU*) might cause continuous flowering, together with reducing the number of runners (Ahmadi et al., 1990; Gaston et al., 2013). However, this locus is not an ortholog of that containing the *FvTFL1* gene in woodland strawberries, indicating the distinct genes in woodland and garden strawberries contributing to the CF phenotype. However, the specific genes of *PFRU* still remain unknown (Lewers et al., 2019; Hytönen and Kurokura, 2020). Recent studies have found that *Arundina graminifolia*, an orchid specie, gives continuous flowering throughout the year (Ahmad et al., 2021). Many essential flowering-related genes have been discovered recently, and a model of regulating continuous flowering has been developed (Ahmad et al., 2021; Li et al., 2021; Ahmad et al., 2022). The model summarizes multiple regulatory pathways, including vernalization, circadian clock, photoperiod, autonomous, and GA pathways. The above studies on continuous flowering can be adopted as a basis for future research on other plants, but the molecular mechanism of continuous flowering remains largely unclear.

Sweet osmanthus (*Osmanthus fragrans* Lour.), a member of the Oleaceae family, is cultivated as a famous ornamental plant in China (Wang et al., 2022). According to flowering characteristics, sweet osmanthus is divided into OF habit, such as ‘Yanhonggui’, and CF habit, like ‘Sijigui’ (Fu et al., 2020). In contrast to OF habit, which only flowers in autumn, CF habit can bloom continuously under favorable environmental conditions (Fu et al., 2020). The obvious flowering differences between OF and CF sweet osmanthus offer excellent materials for studying the regulatory mechanism of continuous flowering. According to Yang (2021), *OfAP1* and *OfTFL1* are the continuous flowering candidate genes in sweet osmanthus, whereas the genetic and molecular basis remains a mystery (Yang et al., 2021). Therefore, the current study analyzes transcriptome data to identify the important genes involved in the continuous flowering of sweet osmanthus. Specific results in this work will provide a guidance for future investigations into the genetic control of continuous flowering in plants.

## Materials and methods

### Plant materials and growth conditions

*O. fragrans* ‘Sijigui’ and ‘Yanhonggui’ used in this study were cultivated in a Pingshan tree nursery (Hangzhou, China, N30°15′14′′N, 119°43′39′′E). The trees used for experiment flowered normally for several years and showed similar growth under natural conditions. For ‘Sijigui’, undifferentiation buds (S0) and differentiation buds (mix of inflorescence primordium differentiation stage and small floret primordium differentiation stage, S1) in winter (flowering in winter) and early summer (blooming in autumn) were collected, and for ‘Yanhonggui’, buds with S0 and S1 stages in early summer (blooming in autumn) were collected, respectively. The collected buds from ‘Sijigui’ and ‘Yanhonggui’ were used for transcriptome sequencing and expression analysis. All materials were saved in liquid nitrogen and immediately refrigerated at -80°C prior. The methods to culture the callus from ‘Sijigui’ were described in a previous research (Zhu et al., 2022). Seeds of *Arabidopsis* (Col-0) were maintained in our laboratory and sown on Murashige and Skoog medium to screen transgenic plants. The seedlings were transferred to a light incubator under a 16-h light/8-h dark photoperiod at 22°C.

### Phenotypic observation

To understand the flowering time and phenotypic changes of sweet osmanthus, the developmental stages of buds in 1 year were observed by stereo microscope and paraffin sections (from 15 April 2020 to 1 April 2021). A total of 10 buds from the three plants were collected every 15 days and stored in FAA fixative (3:1 ratio of 10% ethanol: glacial acetic acid) for paraffin section observation. The method of paraffin section referred to Fan et al. 2018. Samples were sliced to a 10-µm thickness, and an Axio Imager A2 positive fluorescence microscope (Carl Zeiss, Oberkochen, Germany) was used to observe and photograph the development stages of buds. At the same time, the buds were also observed through a microscope.

### Illumina sequencing

**Figure 1:**
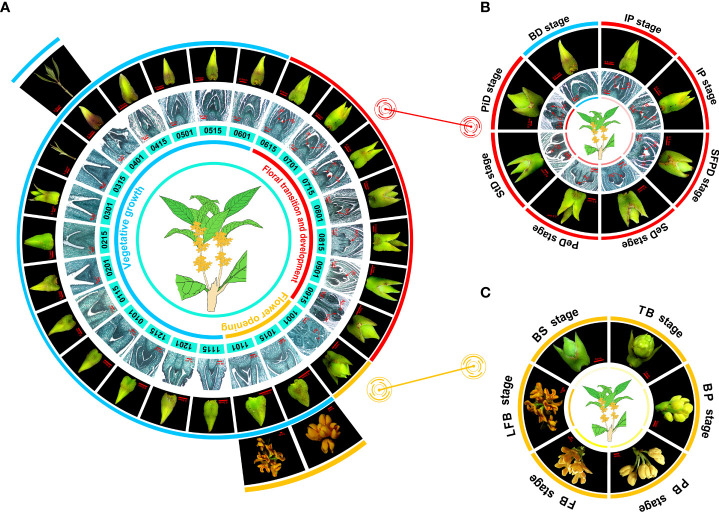
The flowering phenology shifts in *O. fragrans* ‘Yanhonggui’ for 1 year. **(A)** The phenotypic changes of ‘Yanhonggui’ in 1 year. Different colored lines represent different stages of development: blue represents vegetative growth; red means floral transition and development; orange represents flower opening. The numbers below the picture represent dates. For example, 0115 is January 15. **(B)** The floral transition and development process. BD: bud pre-differentiation stage; IP: inflorescence primordium differentiation stage; SFPD: small floret primordium differentiation stage; SeD: sepal differentiation stage; PeD: petal differentiation stage; StD: stamen differentiation stage; PiD: pistil differentiation stage. Due to the obvious change in phenotype, the IP stage was repeated. **(C)** The flower opening process. BS, ball-shaped stage; TB, top bracts stage; BP, bud pedicel stage; PB, primary blooming stage; FB, full blooming stage; LFB, late full blooming stage. Br, Bract; Ip, inflorescence; FP, floret primordium; Se, sepa primordium; Pe, petal primordium; St, stamen primordium; An, anther; Car, carpel.

**Figure 2:**
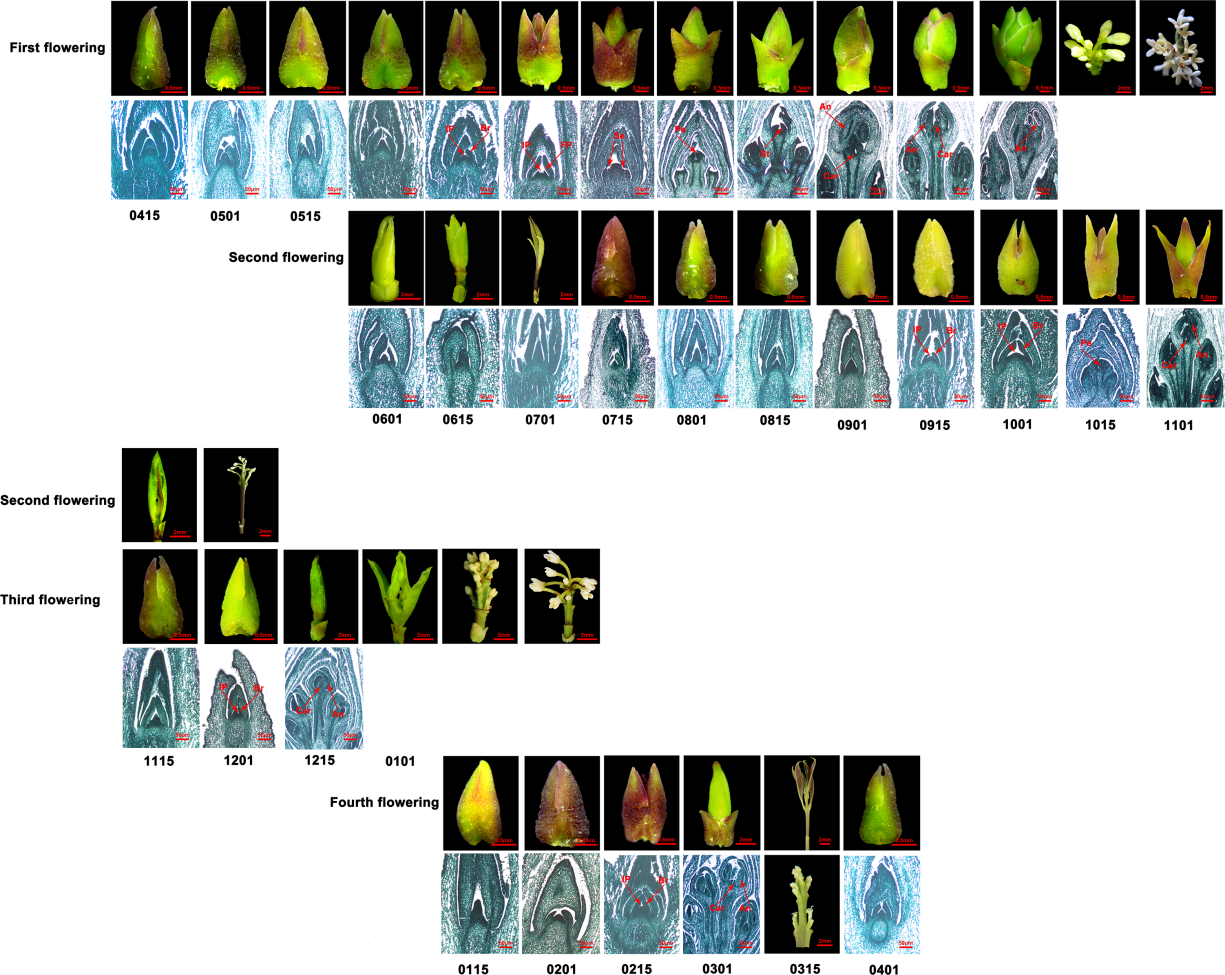
The flowering phenology shifts in *O. fragrans* ‘Sijigui’ for 1 year. First to fourth flowerings respectively represent different flowering processes in 1 year. The numbers below the picture represent dates. For example, 0115 is January 15. Br, bract; Ip, inflorescence; FP: floret primordium; Se, sepa primordium; Pe, petal primordium; St, stamen primordium; An, anther; Car, carpel.

For ‘Sijigui’, buds at S0 and S1 stages in winter were collected on November 15 and December 1, respectively (referring to the third flowering process in **Figure 2**), and the S0 and S1 buds in early summer were collected on June 1 and June 15, respectively (referring to the first flowering process in **Figure 2**). For ‘Yanhonggui’, buds at S0 and S1 stages in early summer were also collected on June 1 and June 15, respectively (referring to the flowering process in **Figure 1A**). RNA from the six tissues was extracted. Prokaryotic mRNA was enriched by eliminating rRNA using the Ribo-Zero™ Magnetic Kit, whereas eukaryotic mRNA was enriched with Oligo(dT) beads (Epicentre). With the use of random primers and fragmentation buffer, the enriched mRNA was reverse transcribed into cDNA. DNA polymerase I, RNase H, dNTP, and buffer were used to create second-strand cDNA. The cDNA fragments were then end repaired and poly(A) added. Illumina sequencing adapters were ligated after they had been purified using a QIAquick PCR extraction kit. Gene Denovo Biotechnology Co. used agarose gel electrophoresis to size-select the ligation products before PCR amplification and Illumina HiSeq™ 4000 sequencing (Guangzhou, China).

### Analysis of differentially expressed genes

To identify differentially expressed genes across samples or groups, the edgeR package (http://www.r-project.org/) was used. We identified genes with a fold change ≥2 and a false discovery rate (FDR) of <0.05 in a comparison as significant differentially expressed genes (DEGs). DEGs were then subjected to enrichment analysis of GO functions and KEGG pathways.

### GO enrichment analysis

Gene Ontology enrichment analysis provided all GO terms that were significantly enriched in DEGs compared with the genome background and filtered the DEGs that correspond to biological functions. Firstly, all DEGs were mapped to GO terms in the Gene Ontology database (http://www.geneontology.org/). Then, gene numbers were calculated for every term, and significantly enriched GO terms in DEGs compared with the genome background were defined by the hypergeometric test. The estimated p-value underwent FDR correction with a threshold of ≤0.05. GO terms that fit these criteria were deemed to be considerably enriched in DEGs.

### Pathway enrichment analysis

KEGG pathway enrichment analysis, comparing DEGs with the backdrop of the entire genome, revealed highly enhanced metabolic or signal transduction pathways. The calculation method is similar to that used in GO analysis above. The estimated p-value underwent FDR correction with a threshold of FDR ≤0.05. Significantly enriched pathways in DEGs were identified as those that met this requirement.

### Quantitative real-time PCR analysis

Specific genes were chosen for expression analysis through qRT-PCR. The buds from the middle and upper regions of the plants were collected on the 1st and 15th of every month in 1 year (total 24 different samples). Since we paid more attention to the new shoot growth and floral transition process, buds from April 15 to June 15 were collected in the first flowering process (**Figure 2**). From July 1 to November 1, buds were collected in the second flowering process. From November 15 to January 1, buds were collected in the third flowering process, and buds from January 15 to April 1 were collected in the fourth flowering process. Total RNA from 24 samples during a year was extracted by the RNAprep Pure Plant Kit (Vazyme, Nanjing, China). cDNA was obtained using HiScript® III Reverse Transcriptase (Vazyme, Nanjing, China). qRT-PCR was performed by the LightCycler 480 qRT-PCR System II (Roche, Basel, Switzerland): SYBR Premix Ex Taq™ 10 µl, up- and downstream primer (10 μmol/l) 0.8 μl, cDNA 2 μl, ddH_2_O 6.4 μl, total 20 µl. Three biological replicates were performed. *OfACT* and *AtACTIN2* (At3g18780) were the reference genes for sweet osmanthus and *Arabidopsis*, respectively.

### Transformation in ‘Sijigui’ callus and *Arabidopsis*

*OfFT* and *OfBFT* were inserted into the pORER4-35S-GFP vector with restriction enzyme cutting sites of *NheI* and *XhoI* (**Supplementary Table S1**). The fusion vector was transformed into GV3101 through a heat shock method. Then, the infection solution was configured to OD_600_ = 0.4–0.6 with the buffer solutions (10 mM MES, 10 mM MgCl_2_, and 0.1 mM AS) and kept standing at room temperature for 3 h. The callus of *O. fragrans* was added to the infection solution and cultured at 28°C for 0.5 h under dark conditions. The callus was washed for four times with ddH_2_O and cultured in symbiotic mediums with 3 days under dark conditions. After that, the callus was saved in a -80°C refrigerator for further analysis. The constructed vectors were also introduced into *Arabidopsis* Col-0. The MS medium containing 50 mg/l kanamycin was used to screen the transgenic plants. We obtained 18 and 15 independent transgenic lines of *OfFT* and *OfBFT*, respectively, and homozygous transgenic lines from T3 were used for further research. For expression analysis, the entire shoots of T3 homozygous transgenic lines of *OfFT* and *OfBFT* were collected at 14 and 20 days, respectively, after planting and subjection to qRT-PCR analysis.

### Yeast one-hybrid assay

Fragments of the *OfFT* and *OfBFT* promoter were cloned into the pHis2 vector, and pHis2-*proOfFT*/*OfBFT* were transformed into Y187 to screen the suitable resistance concentrations of 3AT. The suitable concentrations were used to screen ‘Sijigui’ cDNA library and interaction validation. A yeast one-hybrid system was performed using the Matchmaker™ Gold Yeast Two-Hybrid System from Clontech as recommended by the manufacturer.

### Dual-Glo® luciferase assay

The interaction between *Of*SPL8 protein and the promoter of *OfFT* and *OfBFT* was verified by the Dual-Glo® Luciferase Assay. *OfSPL8* was cloned into the pORER4-35S-GFP vector, and the promoters of *OfFT* and *OfBFT* were cloned into pGreen0800II-LUC. All vectors were transformed into GV3101 (pSoup-p19). Then, 35S::*OfSPL8* and *proOfFT-*LUC were coinjected into the leaves of tobacco, together with 35S::*OfSPL8* and *proOfBFT-*LUC. The activities of fluorescein enzymes were measured after 3 days. The activities of firefly luciferase (LUC) and renilla luciferase (REN) were measured using the GloMax® multifunctional instrument (Promega, USA).

## Results

### Flowering phenology shifts of OF habit in sweet osmanthus

To disclose the OF habit flowering characteristics in sweet osmanthus, *O. fragrans* ‘Yanhonggui’ was selected and buds at the upper half of a branch were continuously taken for follow-up observation. The microscope and paraffin sections were used, and the observation continued throughout the year. We found that the annual growth cycle of ‘Yanhonggui’ could be divided into three periods: vegetative growth, floral transition and development, and flower opening (**Figure 1A**). After the flowers had faded in the autumn, vegetative growth commenced and lasted until the buds entered the floral transition in the next early June (early summer). Additionally, it was discovered that during vegetative development, buds grew slowly with no significant changes in phenotype during the whole winter but quickly generated new shoots in the spring. The buds in new shoots would continue to grow in spring and enter floral transition in early summer and bloom in autumn (**Figure 1A**). According to paraffin sections, the floral transition and development period occurred from June to September. The whole period was further divided into seven stages: bud pre-differentiation stage (BD), inflorescence primordium differentiation stage (IP), small floret primordium differentiation stage (SFPD), sepal differentiation stage (SeD), petal differentiation stage (PeD), stamen differentiation stage (StD), and pistil differentiation stage (PiD). Due to the obvious change in phenotype, the IP stage was repeated (**Figure 1B**). Based on the phenotypic of the buds, it suggested that during the floral development process, the apical bracts of the buds were dehisced gradually, together with the expansion of the whole buds. Interestingly, although the buds had finished the floral development process (having well-developed pollen grains), they could not bloom immediately until when the ambient temperature was cooler in autumn. The blooming time of ‘Yanhonggui’ is not fixed each year because of the variable cooling time in the fall. Our previous research revealed that ambient temperature had significant effects on flower opening in ‘Yanhonggui’ (Fu et al., 2020). Therefore, we believed that the actual blooming time in ‘Yanhonggui’ depends more on the flower opening than the floral transition. Similar to the floral transition and development period, the flower opening period, based on phenotypic differences, was also made up of different stages. Here, we presented the six stages with the most significant phenotypic changes to introduce the changes of phenotype: ball-shaped stage (BS), top bracts stage (TB), bud pedicel stage (BP), primary blooming stage (PB), full blooming stage (FB), and late full blooming stage (LFB) (**Figure 1C**).

### Flowering phenology shifts of CF habit in sweet osmanthus

Compared with ‘Yanhonggui’, although ‘Sijigui’ had a similar floral transition and development process in early summer and flower opening process in autumn, it could bloom constantly from October to the following June. According to the observations using microscope and paraffin sections, new shoot growth and floral development occurred concurrently and mainly had four times in 1 year, with the most vigorous flowering in autumn (**Figure 2**). Paraffin sections revealed that the main floral transition events lasted about 2.5 months (first flowering), 1.5 months (second flowering), 1 month (third flowering), and 1.5 months (fourth flowering), respectively. The floral transition processes began in early June, mid-September, early December, and early February and ended in late August, early November, early January, and mid-March, respectively. In addition to the differences in the time required for flower bud differentiation, ‘Sijigui’ had discrepancies in the structure of inflorescences in different seasons. It produced cymose inflorescence in autumn and racemose inflorescence in winter and spring, which had a pedicure at the base of flowers (**Figure 2**). The results suggested that compared with autumn, the molecular mechanism of flowering regulation might have a difference in winter and spring in ‘Sijigui’.

We also discovered that numerous buds generated in early July were unable to undergo floral transition throughout the whole summer until the cooler autumn (**Figure 2**). Ambient temperature had a vital role in flowering regulation in sweet osmanthus (Zhu et al., 2019). We hypothesized that ambient temperature might be involved in continuous flowering regulation of CF habit, and high temperatures in the summer impeded floral transition and led to few or no blooms of “Sijigui” in the summer.

### Illumina sequencing and analysis of DEGs

**Figure 3:**
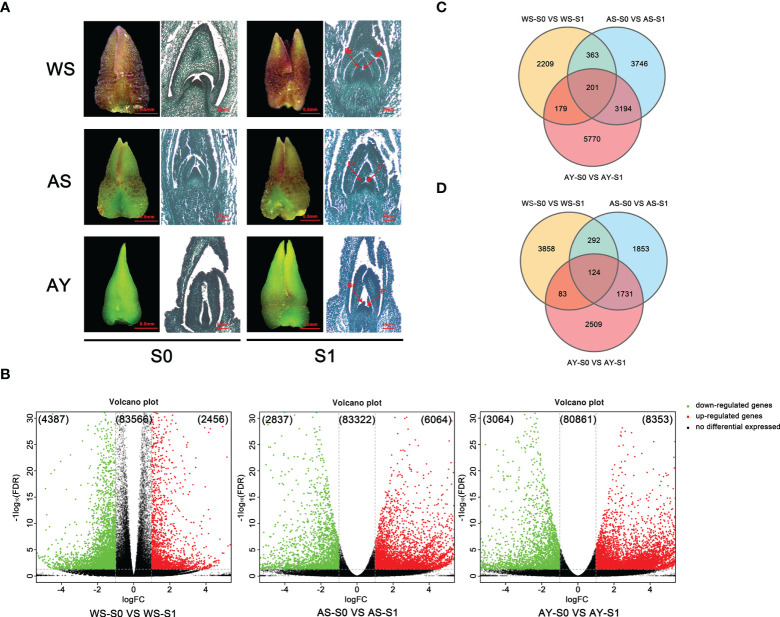
Comparative analysis of DEGs during the floral transition process in OF and CF habits. **(A)** Samples for transcriptome sequencing analysis. WS: buds blooming in winter from ‘Sijigui’; AS: buds blooming in autumn from ‘Sijigui’; AY: buds blooming in autumn from ‘Yanhonggui’. S0: undifferentiation buds; S1: differentiation buds (mix of inflorescence primordium differentiation stage and small floret primordium differentiation stage). Br: bract; Ip: inflorescence; FP: floret primordium. **(B)** The expression trends of DEGs in the three comparisons. Green circles: downregulated genes; red circles: upregulated genes; black circles: non-differentially expressed genes. **(C)** Venn diagram of the number of upregulated genes among the three comparisons. **(D)** Venn diagram of the number of downregulated genes among the three comparisons.

To further study the possible regulatory mechanism of continuous flowering in sweet osmanthus, we collected three biological replicate samples of S0 and S1 from ‘Sijigui’ in winter and early summer and S0 and S1 from ‘Yanhonggui’ in early summer for transcriptome sequencing (**Figure 3A**). It generated about 966.8 million reads from six development stages. Each sample produced about 6.2–9.6 Gb clean data with an average of 26.2 million clean reads (**Supplementary Table S2**). We filtered 94,529 differentially expressed unigenes (DEGs), which were annotated using NR, SWISSPORT, KOG, and KEGG databases. The covering annotation data were 48.6%, 31.86%, 27.30%, and 21.23%, respectively (**Supplementary Table S3**).

Detailed comparative analysis of the DEGs was performed in three comparisons, namely, WS-S0 vs. WS-S1, AS-S0 vs. AS-S1, and AY-S0 vs. AY-S1 (P value ≤0.01 and |log2Ratio| ≥ 1). We found that 4,387 and 2,456 unigenes were down- or upregulated, respectively, in the WS-S0 vs. WS-S1 comparison, whereas the unigenes down- or upregulated in the AS-S0 vs. AS-S1 combination were 2,837 and 6,064, respectively. Meanwhile, 3,064 and 8,353 unigenes in the AY-S0 vs. AY-S1 comparison were down- or upregulated, respectively (**Figure 3B**). In summary, upregulated unigenes were more than downregulated unigenes during the three comparisons. In addition, only 201 DEGs were all upregulated in three comparisons, and 2,209, 3,746, and 5,570 DEGs were upregulated in the WS-S0 vs. WS-S1, AS-S0 vs. AS-S1, and AY-S0 vs. AY-S1 comparisons, respectively (**Figure 3C**). Simultaneously, 124 DEGs were downregulated in three comparisons, and 3,858, 1,853, and 2,509 DEGs in the WS-S0 vs. WS-S1, AS-S0 vs. AS-S1, and AY-S0 vs. AY-S1 comparisons were downregulated, respectively (**Figure 3D**).

### GO enrichment and KEGG pathway analysis of DEGs

In all three comparisons, ‘metabolic process’, ‘cellular process’, and ‘catalytic activity’ were the most significantly enriched GO terms in the biological process. In the cellular component, ‘cell’, ‘cell part’, and ‘membrane’ were highly enriched in the WS-S0 vs. WS-S1 comparison, whereas ‘cell’, ‘cell part’, and ‘organelle’ were significantly enriched in the other two comparisons. In the molecular function, ‘Catalytic activity’ and ‘Binding’ were obviously enriched among the three comparisons (**Supplementary Figure S1**). There were 20,065 unigenes mapped into 132 KEGG pathways, with 121, 128, and 131 KEGG metabolic pathways in the WS-S0 vs. WS-S1, AS-S0 vs. AS-S1, and AY-S0 vs. AY-S1 comparisons, respectively (**Supplementary Table S4**).

### Identification of flowering-related genes

**Figure 4:**
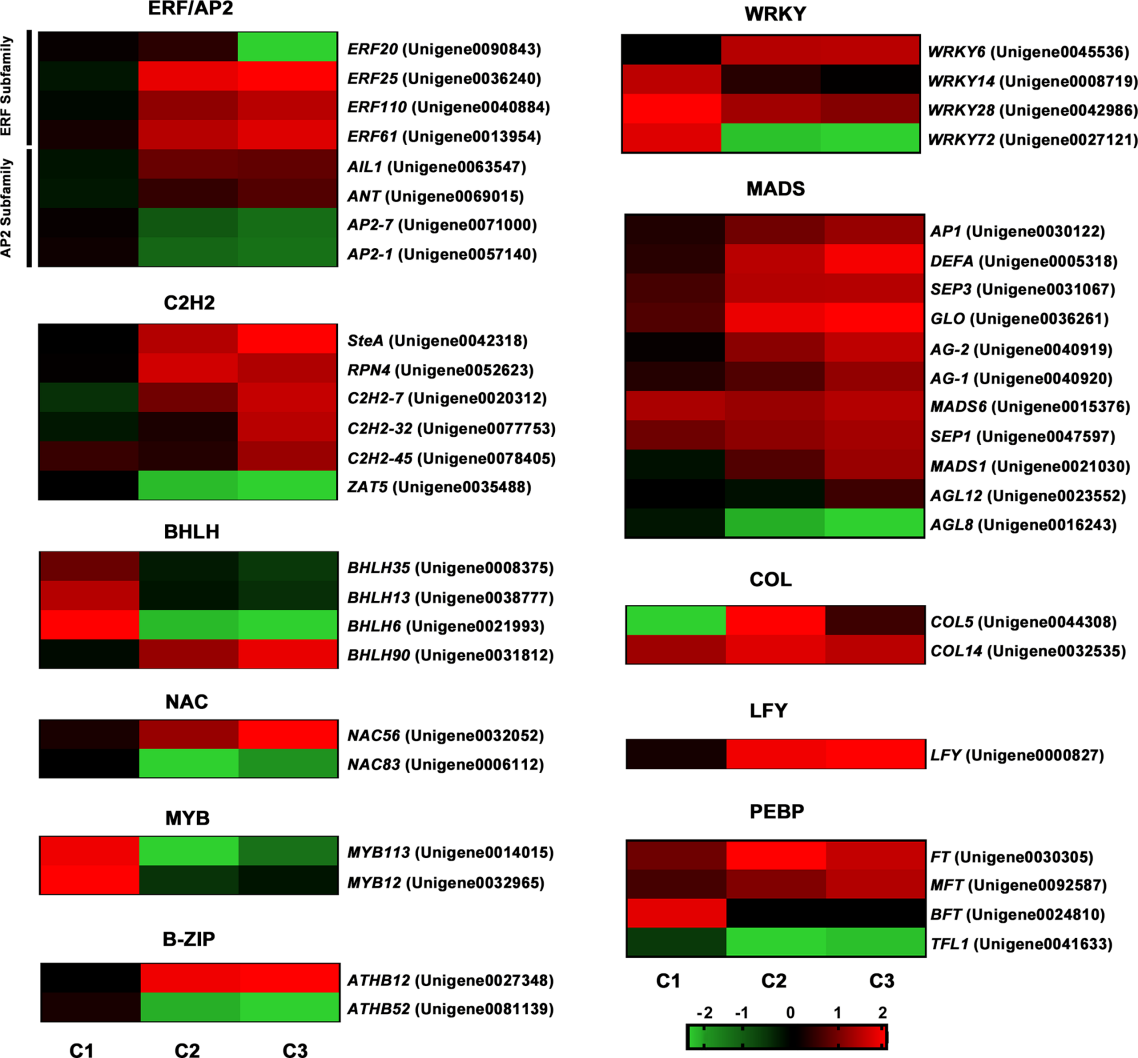
Comparative analysis of DEGs during the floral transition process in OF and CF habits. Red means the upregulation, and green means the downregulation. C1, C2, and C3 represent WS-S0 vs. WS-S1, AS-S0 vs. AS-S1, and AY-S0 vs. AY-S1 comparisons.

Our data identified 1,990 transcription factors (TFs) from 58 TF families and found some flowering-related families, including the ERF/AP2, C2H2, BHLH, NAC, MYB, WRKY, bZIP, COL, MADS and PEBP families (**Supplementary Table S5**). The expression patterns of the TFs from the above families were analyzed (**Figure 4**). In the ERF/AP2 family, three of them (*ERF25*, *ERF110*, *ERF61*) were significantly upregulated in comparison AY-S0 vs. AY-S1(C3), and *ERF20* was downregulated in the C3 comparison, whereas the expression of two genes (*AP2-1*, *AP2-7*) was decreased in the AS-S0 vs. AS-S1(C2) and C3 comparisons, accompanied by induction of two genes (*AIL1*, *ANT*). Six genes from the C2H2 family were obviously expressed. Three genes (*SteA*, *RPN4*, *C2H2-7*) were increased in C2 and C3 comparisons, and one gene (*ZAT5*) was decreased. Two genes (*C2H2-32*, *C2H2-45*) were just upregulated in the C3 comparison. In the BHLH family, the expressions of *BHLH35*, *BHLH13*, and *BHLH6* were promoted in the WS-S0 vs. WS-S1(C1) comparison and inhibited in the C2 and C3 comparisons. The accumulation of *BHLH90* was increased in the C2 and C3 comparisons. Only two genes in the NAC family had a significant expression, in which *NAC56* was inhibited and *NAC83* was promoted respectively in the C2 and C3 comparisons, whereas in the MYB family, two genes (*MYB113* and *MYB12*) were all downregulated in the C2 and C3 comparisons. It was found that in the WRKY family, *WRKY28* was upregulated in all three comparisons and *WRKY6* was upregulated in the C2 and C3 comparisons. *WRKY14* and *WRKY72* were accumulated in the C1 comparison, and *WRKY72* showed a significant reduction in the C2 and C3 comparisons. In the B-ZIP family, *ATHB12* and *ATHB52* exhibited opposite expression patterns in both C2 and C3 comparisons. *ATHB12* was increased in the C2 and C3 comparisons and *ATHB52* was decreased. Eleven genes in the MADS family had an obvious expression, and seven of them (*AP1*, *DEFA*, *SEP3*, *GLO*, *AG-1*, *MADS6*, *SEP1*) were upregulated in three comparisons. *AG-2* and *MADS1* expressions were increased in the C2 and C3 comparisons, whereas *AGL8* was decreased in the same comparisons. *AGL12* was only upregulated in the C3 comparison. In the COL family, the expression of *COL14* was promoted in three comparisons. *COL5* was increased in the C2 comparison and decreased in the C1 comparison. Four genes with a significant expression in the PEBP family were found, and two genes (*FT* and *MFT*) were upregulated in all three comparisons, especially in the C2 and C3 comparisons. *BFT* has a specific high expression in the C1 comparison. *TFL1* was downregulated in three comparisons. In addition to the above families, we identified an *LFY* homologous gene, which was highly expressed in C2 and C3 comparisons, without obvious change in the C1 comparison.

### Expression patterns of PEBP family genes correlated with CF habit in ‘Sijigui’

**Figure 5:**
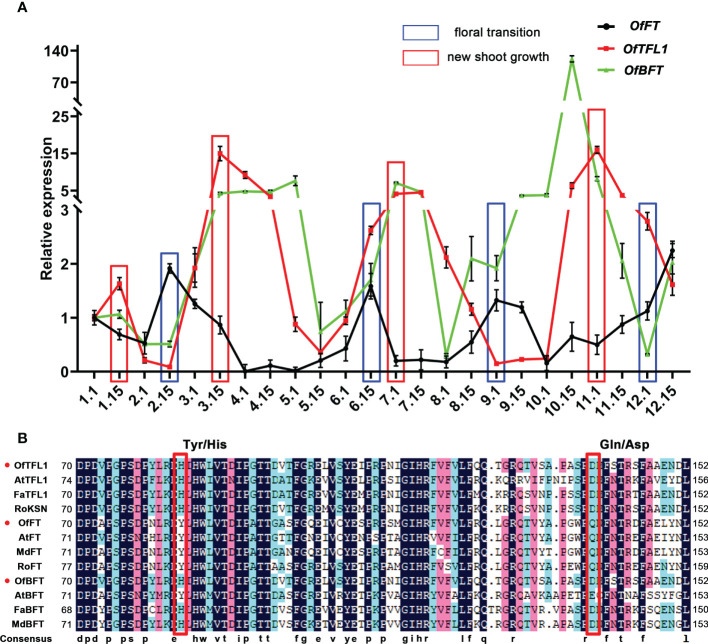
Isolation and expression analysis of *OfFT*, *OfBFT*, and *OfTFL1* in OF and CF habits. **(A)** Expression patterns of *OfFT*, *OfBFT*, and *OfTFL1* in 1 year. Red boxes represent the time new shoots occur. Blue boxes represent the time of floral transition. Means ±SD, n = 3. **(B)** Multiple amino acid sequence alignment of TFL1 and FT proteins in different species. Red box indicates Tyr/His and Gln/Asp that differed between TFL1 and FT proteins.

Previous studies indicated that several genes from the PEBP family had important roles in CF habit, especially the homologous genes of *TFL1* and *FT* (Koskela et al., 2012; Ahmad et al., 2022). Here, we focused on the homologous genes of *FT* (Unigene0030305, *OfFT*), *TFL1* (Unigene0041633, *OfTFL1*), and *BFT* (Unigene24810, *OfBFT*), which were significantly expressed in three comparisons, whereas there was no difference in the DNA sequence of the three genes in OF and CF habits (**Supplementary Figure S2A**). We further analyzed their expression patterns during 1 year in ‘Sijigui’ (**Figure 5A**). It showed that the three genes had distinct expression characteristics. They all had four different expression peaks during a year, and the expression patterns of *OfTFL1* and *OfBFT* were similar but opposite to *OfFT*. Moreover, referring to the flowering phenology shifts of ‘Sijigui’, each new shoot growth was accompanied by a high expression of *OfTFL1* and *OfBFT* and a low expression of *OfFT*, whereas *OfFT* was upregulated during the floral transition processes, with downregulation of *OfTFL1* and *OfBFT*. The results suggested that in ‘Sijigui’, both *OfTFL1* and *OfBFT* might play important roles in new shoot growth, and *OfFT* might be involved in the floral transition process. Amino acid sequence alignment showed that *OfTFL1* contained two conservative residues (His84 and Asp139), which could distinguish *TFL1* homologous genes from *FT* homologous (**Figure 5B**). Moreover, *OfBFT* had the same conservative residues as with *OfTFL1*. Phylogenetic analysis suggested that *OfTFL1*, *OfBFT*, and *OfFT* were closely related to the *TFL1* subfamily, *BFT* subfamily, and *FT* subfamily, respectively (**Supplementary Figure S2B**).

### *OfFT* functioned as a flowering promoting gene in ‘Sijigui’ and *Arabidopsis*

**Figure 6:**
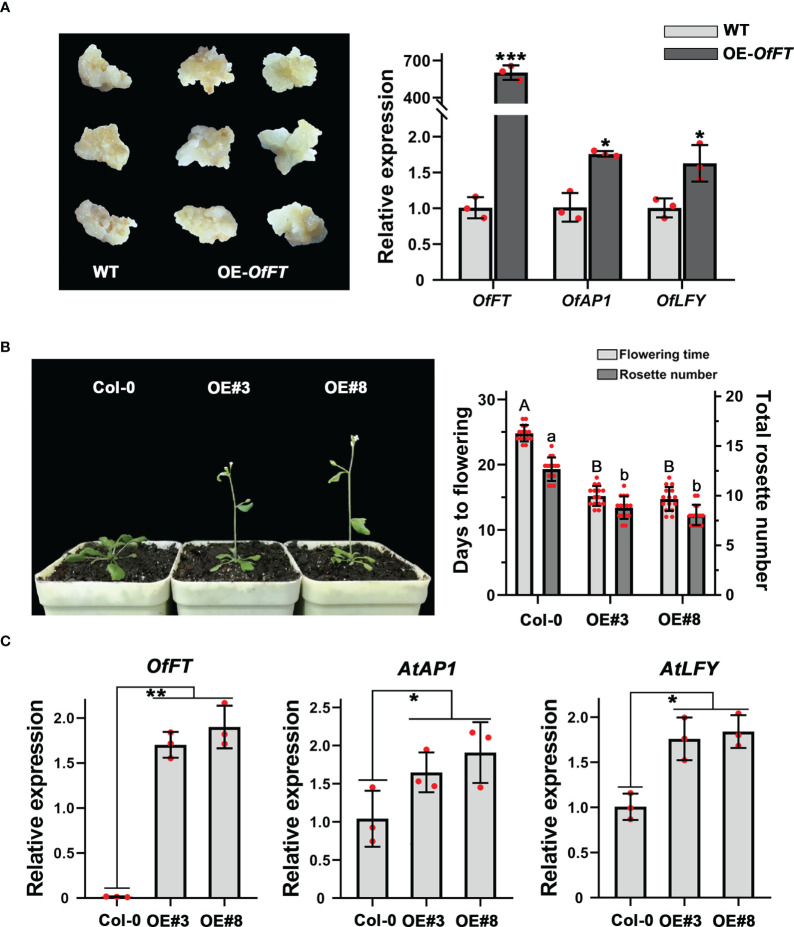
*OfFT* functioned as a flowering activator in both *O. fragrans* and *Arabidopsis*. **(A)** The expression levels of flowering-related genes in overexpression ‘Sijigui’ callus. Data were the means ±SD, n = 3, Student’s t test, *p < 0.05 and ***p < 0.001. **(B)** Phenotypic analyses of transgenic Arabidopsis plants overexpressing *OfFT*. Uppercase letters represent significance of flowering time compared with mock-treated control. Lowercase letters represent significance of rosette number compared with mock-treated control. Means ± SD, n = 3, *p < 0.05, **p < 0.01. For **Figure 6**: **(C)** The expression levels of flowering-related genes in transgenic plants. Means ±SD, n = 3. *p < 0.05, **p < 0.01.

To elucidate the biological function of *OfFT*, we overexpressed it in the callus of ‘Sijigui’, driven with the cauliflower mosaic virus (CaMV) 35S promoter, and measured the expression levels of *OfFT* and some flowering-related genes. It showed that compared with WT, *OfFT* was highly expressed in the callus (**Figure 6A**). According to previous studies, *FT* could promote flowering through upregulating the expression of *AP1* and *LFY* (Carmona et al., 2007; Jeon et al., 2011). Here, the accumulation of *OfAP1* and *OfLFY* in *OfFT*-overexpressed callus was also detected, and it showed that both the genes were induced (**Figure 6A**). We also verified the role of *OfFT* in flowering regulation in wild-type *Arabidopsis* (Col-0). Homozygous lines of the T3 generation were chosen for the phenotypic observation. The transgenic plants displayed early flowering phenotypes with less rosette leaves than wild-type plants under long-day conditions (**Figure 6B**). The entire shoots of T3 homozygous transgenic lines of *OfFT* were collected at 14 days after planting, and the expression levels of *AtFT*, *AtAP1* and *AtLFY* in transgenic plants were investigated. Compared with Col-0, it indicated that the expressions of *AtAP1* and *AtLFY* were increased in transgenic plants, whereas the expression levels of *AtFT* had no significant change (**Figure 6C**, **Supplementary Figure S3A**). All results showed that *OfFT* functioned as a positive regulator of flowering by inducing the expression of flowering-related genes.

### *OfBFT* functioned as a flowering suppressor in ‘Sijigui’ and *Arabidopsis*

**Figure 7:**
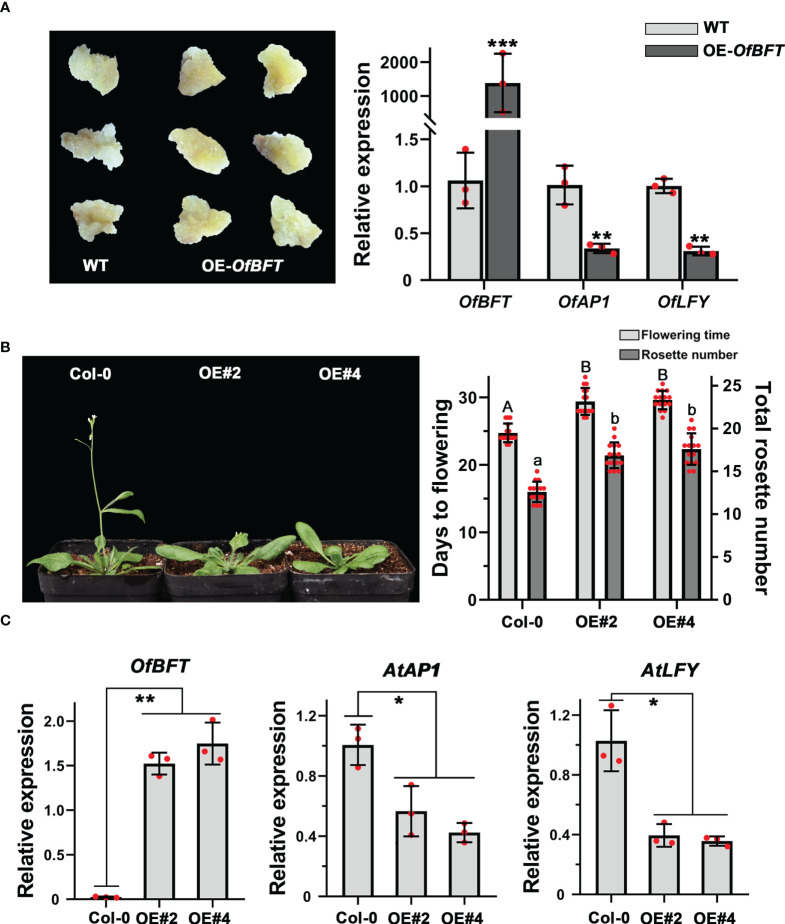
*OfBFT* functioned as a flowering suppressor in both *O. fragrans* and *Arabidopsis*. **(A)** The expression levels of flowering-related genes in overexpression ‘Sijigui’ callus. Data were the means ±SD, n = 3, Student’s t test, **p < 0.01 and ***p < 0.001. **(B)** Phenotypic analyses of transgenic Arabidopsis plants overexpressing *OfBFT*. Uppercase letters represent significance of flowering time compared with mock-treated control. Lowercase letters represent significance of rosette number compared with mock-treated control. Means ± SD, n = 3, *p < 0.05, **p < 0.01. **(C)** The expression levels of flowering-related genes in transgenicplants. Means ±SD, n = 3. *p < 0.05, **p < 0.01.

To reveal the role of *OfBFT* in flowering regulation, we also overexpressed it in the callus of ‘Sijigui’ and *Arabidopsis* and performed qRT-PCR to confirm the transformation efficiency. It showed that *OfBFT* was effectively accumulated in the callus. In flowering time control of *Arabidopsis*, *BFT* is functionally redundant with *TFL1*, which determines the floral transition by inhibiting the expression of *LFY* and *AP1* (Ryu et al., 2014). In this study, overexpression of *OfBFT* resulted in the downregulated expression of *OfAP1* and *OfLFY* (**Figure 7A**). In *Arabidopsis*, the ectopic expression of *OfBFT* affected the reproductive development process with a late-flowering phenotype and more rosette leaves under long-day conditions (**Figure 7B**). We then also studied the expression levels of the vital flowering-related genes *AtBFT*, *AtAP1*, and *AtLFY*. The entire shoots of T3 transgenic lines were collected at 20 days after planting, and the mRNA levels were analyzed. Compared with Col-0, it demonstrated that the expression of *AtBFT* was not obviously changed, whereas *AtAP1* and *AtLFY* were significantly decreased in transgenic plants (**Figure 7C**, **Supplementary Figure S3B**). All results showed that *OfBFT* was a flowering suppressor by reducing the accumulation of flowering activating genes.

### *OfSPL8* could bind to the promoter of *OfFT* and *OfBFT* to regulate their expression

**Figure 8:**
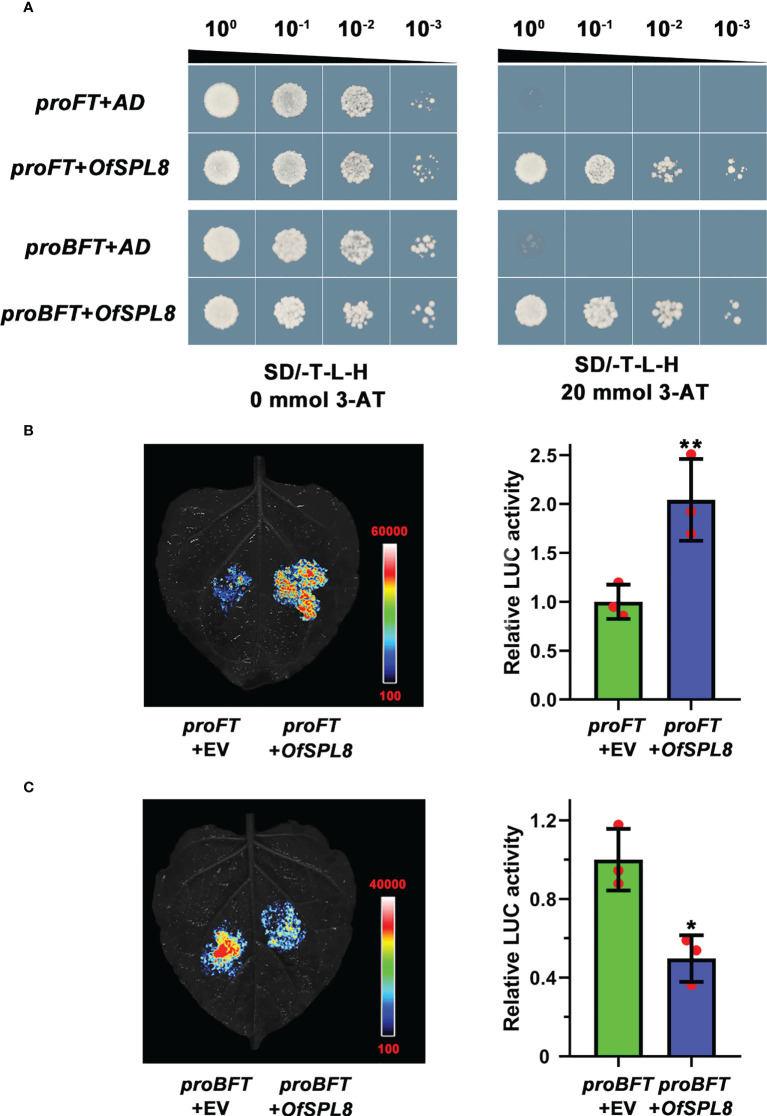
The binding between *OfSPL8*, *proOfFT*, and *proOfTFL1*. **(A)** Yeast one-hybrid analysis of *proOfFT*, *proOfBFT*, and *OfSPL8*. **(B)** Dual-LUC analysis of *proOfFT* and *OfSPL8*. Student’s t test means ± SD, n = 3, **p < 0.01. **(C)** Dual-LUC analysis of *proOfBFT* and *OfSPL8*. Student’s t test means ±SD, n = 3, *p < 0.05.

A yeast one-hybrid assay was performed to screen the potential upstream transcription factors through the promoter of *OfFT* and *OfBFT*, respectively. The ‘Sijigui’ cDNA library was built previously in our lab. Interestingly, we found that *OfSPL8* was a common upstream transcription factor of *OfFT* and *OfBFT*. Because of the existence of false positives, the binding among *OfSPL8*, *proOfFT*, and *proOfBFT* was investigated through yeast one-hybrid and dual-luciferase (dual-LUC) assay. It showed that both the cells harboring *OfSPL8* in the pGADT7 recombinant vector and *proFT* or *proBFT* in the pHis2 recombinant vector were grown normally on TDO (20 mM 3-AT) solid medium (**Figure 8A**). The results of the dual-LUC assay suggested that *OfSPL8* could significantly induce the transcriptional activity of the *OfFT* promoter (**Figure 8B**) and obviously inhibit the activity of the *OfBFT* promoter (**Figure 8C**). These results revealed that *OfSPL8* could bind to the promoter of *OfFT* and *OfBFT* to regulate their expression in ‘Sijigui’.

## Discussion

Flowering, transitioning from vegetative growth to reproductive growth, is an important life activity for plants (Chen D. et al., 2018; Chen Z. et al., 2018). Most plants flower once a year, and only a few plants bloom in multiple seasons, such as *Osmanthus fragrans* var. ‘Sijigui’, *Jasminum sambac*, *Fuchsia hybrida*, *Rosa chinensis*, and *Citrus limon* (Yang et al., 2021). Continuous flowering (CF) is one of the most important horticultural traits for ornamental plants, although there is only limited scientific understanding about this phenomenon. Based on current studies, not only flowering habit but also some important development processes have differences between OF and CF habits, including the length of juvenile stage and inflorescence architecture (Iwata et al., 2012).

In rose, the primary shoots of OF plants remained to have indeterminate growth and only flowered on axillary secondary shoots (Vries, 1976), whereas in CF roses, plants had a short juvenile stage and bloomed rapidly after seed germination. Moreover, all shoots had determinate growth with a terminal inflorescence in CF roses (Vries, 1976). In OF woodland strawberry, the terminal meristem differentiated into an inflorescence, and the axillary meristems maintained vegetative development, which might develop into runners (Koskela et al., 2018), whereas in CF habit, runner growth was inhibited and the axillary meristems could differentiate into inflorescences under favorable conditions (Koskela et al., 2018). A CF mutant, *super long blooming1* (*slb1*), was found in *Liriodendron chinense* (Sheng et al., 2021). The *slb1* mutant had a prolonged flowering phase and performed floral transition and flowering at the same season (Sheng et al., 2021). In OF habit of sweet osmanthus, flower buds differentiated in summer and bloomed in autumn. After flowering, some vegetative buds grew slowly and then formed new shoots in the next spring (**Figure 1**). Although CF habit had the same flowering process in autumn as OF habit, it had continuity of vegetative growth and formed favorable buds for the following floral transition and flowering (**Figure 2**). Additionally, like CF roses, CF sweet osmanthus also had a terminal inflorescence on new shoots. According to the difference between the OF and CF sweet osmanthus, the ability to induce new shoot growth might be closely related to continuous flowering habit.

Many TF families are involved in floral transition and flowering time regulation (Chen D. et al., 2018). Here, we identified some TFs from several flowering-related families, including the ERF/AP2, C2H2, BHLH, NAC, MYB, WRKY, bZIP, COL, MADS, and PEBP families. The MADS, ERF/AP2, and C2H2 families were the top three families with the most differential expression TFs in our study (**Figure 4**). In flowering plants, MADS-box genes are master regulators of the floral transition process (Kennedy et al., 2018). We identified that 11 genes in the MADS family had an obvious expression among the three comparisons. *AP1*, *DEFA*, *SEP3*, *GLO*, *AG-1*, *MADS6*, and *SEP1* were upregulated in all three comparisons, and *AG-2* and *MADS1* expressions were increased in the C2 and C3 comparisons, whereas A*GL12* was only upregulated in the C3 comparison. In *Arabidopsis*, *AtAP1* was a flowering activator and functioned as a downstream of the *AtFT* gene (Wang et al., 2021). *AtSEPs* and *AtAG* were members of the ABCDE genetic model, which participated in floral organ identity (Irish, 2017). In sweet cherries, PavSEP and PavAP1 proteins also interacted with PavSVP to regulate floral transition (Wang et al., 2021). The ERF/AP2 family is one of the largest TF families in the plant kingdom, which is widely involved in the plant development process in *Arabidopsis*, rice, and barley (Sharman, 1994; Mehrnia et al., 2013; Ren et al., 2013). In our study, we found that eight genes from the ERF/AP2 family (four in the ERF subfamily and four in the AP2 subfamily) had a significant expression (**Figure 4**). In rice, several genes from the C2H2 family participate in controlling flowering time (Wu et al., 2019), whereas six genes having different expression patterns were found to have obvious expression among three comparisons in sweet osmanthus. It indicated the different roles of genes in the flowering regulation of OF and CF habits. The BHLH family is the second largest transcription factor family in plants, and in *Arabidopsis*, *BHLH48* and *BHLH60* regulated flowering under long-day conditions through the GA pathway (Li et al., 2017). The expressions of *BHLH35*, *BHLH13*, and *BHLH6* were just promoted in the C1 comparison and inhibited in the C2 and C3 comparisons, suggesting that the three genes might be involved in the floral transition process in winter of CF habit (**Figure 4**). In sweet cherry, woodland strawberry, and *Arabidopsis*, the NAC, MYB, and WRKY families played roles during the floral transition process (Villar et al., 2020; Lei et al., 2020). In our study, only two genes in both the NAC and MYB families had a significant expression, together with four genes in the WRKY family. In the COL family, the expression of *COL14* was promoted in three comparisons, whereas *COL5* was decreased in the C1 comparison. In CF habit rose, RcCOL4 and RcCOL5 were in response to different photoperiods to regulate continuous flowering (Lu et al., 2020). Moreover, a *LFY* homologous gene was highly expressed in C2 and C3 comparisons. Similar to *AtAP1*, *AtLFY* was also involved in flowering regulation and floral organ identity (Wang et al., 2021).

The PEBP family is involved in regulating flowering time and plant architecture (Karlgren et al., 2011). In plants, the PEBP family can be divided into three main clades: *FT-like*, *TFL1-like*, and *MFT-like* subfamilies (Wickland et al., 2015). *FT* and *TWIN SISTER OF FT* (*TSF*) are two members of the *FT-like* subfamily, which acts as a vital signaling molecule to promote flowering (Cao et al., 2016). *FT-like* genes, identified in many species, were mainly accumulated in leaves and then delivered to the buds to control the switch from vegetative growth to reproductive growth (Corbesier et al., 2007). Additionally, in the CF mutant of *Liriodendron chinense*, a unique *FT* splicing variant with intron retention specific to *slb1* mutants represents the vital role of the *FT* gene in CF habit (Sheng et al., 2021). Unlike *FT-like* genes, *TFL1* and *BFT* are classified into the *TFL1-like* clade and are usually known as flowering repressors (Wickland and Hanzawa, 2015). It has also been revealed that *TFL1* in various species function in regulating seed development and inflorescence development and affecting flowering habit (Koskela and Hytönen, 2018; Zhang et al., 2020). This study identified the homologous genes of *FT*, *TFL1*, and *BFT* in both OF and CF habit, whereas the DNA sequence had no difference (**Figure 4**, **Supplementary Figure S2A**). Further studies showed that the expression patterns of the three genes were related to the continuous flowering habit of sweet osmanthus (**Figure 4**). In both rose and woodland strawberries, *TFL1* homologues are good candidate genes for CF habit (Hurst, 1941; Koskela et al., 2012). In kiwifruit, *Acbft* lines displayed an ever-growing phenotype and increased branching, suggesting the role of *BFT* in new branch growth (Herath et al., 2022). Here, the functions of *OfFT* and *OfBFT* were analyzed in ‘Sijigui’ and *Arabidopsis*. The results showed that *OfFT* was a flowering promoting gene in both ‘Sijigui’ and *Arabidopsis* by increasing the expression of *AP1* and *LFY* genes (**Figure 6**), whereas *OfBFT* functioned as a flowering suppressor through downregulating the expressions of *AP1* and *LFY* (**Figure 7**). Interestingly, *OfSPL8* was also found as a common upstream transcriptional regulator of *OfFT* and *OfBFT* and was involved in regulating their expression (**Figure 8**). It indicated the important role of *OfSPL8* in CF habit of sweet osmanthus. *SPLs* have a beneficial function in plant floral transition and inflorescence development through regulating the flower meristem identity gene (Unte, 2003; Zhang et al., 2007). In *Arabidopsis*, *SPLs* are key components of the aging pathway in regulating the transition from plant vegetative growth to reproductive development (Wang, 2014). *SPLs* also act as activators of flowering by regulating flower meristem identity genes in *Arabis alpina* (Akhter et al., 2022).

In conclusion, these findings first revealed the flowering phenology shifts in OF and CF habits of sweet osmanthus. The role of several floral integration genes in CF habit were also analyzed. Our work might provide a new light on the regulatory pathway of continuous flowering.

## Data availability statement

The data presented in the study are deposited in online repositories. The names of the repository/repositories and accession number(s) can be found below: https://www.ncbi.nlm.nih.gov/, SAMN31364653.

## Author contributions

HZ, BD, and QF designed the research. QW, GG, XC, XL, YW, and SZ performed the experiments. QW, GG XC, and XL analyzed the data. QW and GG wrote the manuscript. HZ revised the manuscript. All authors contributed to the article and approved the submitted version.

## Funding

This research was supported by the National Natural Science Foundation of China (Grant Nos. 32072615 and 31902057), the major scientific and technological project of Zhejiang Province (2021C02071), and the molecular mechanism of *FT/TFL1*-mediated floral transition in response to gibberellin in *Osmanthus* ‘Sijigui’ (32201617).

## Acknowledgments

We would like to thank Wei Zhou (Zhejiang Chinese Medical University) for providing the pHis2 and pGreen0800-LUC plasmids.

## Conflict of interest

The authors declare that the research was conducted in the absence of any commercial or financial relationships that could be construed as a potential conflict of interest.

## Publisher’s note

All claims expressed in this article are solely those of the authors and do not necessarily represent those of their affiliated organizations, or those of the publisher, the editors and the reviewers. Any product that may be evaluated in this article, or claim that may be made by its manufacturer, is not guaranteed or endorsed by the publisher.
