# Supplementary figures and images for: Genetic studies on continuous flowering in woody plant Osmanthus fragrans
Source: Front Plant Sci. 2022 Nov 3;13:1049479. doi: 10.3389/fpls.2022.1049479 (PMC9671776; doi:10.3389/fpls.2022.1049479)

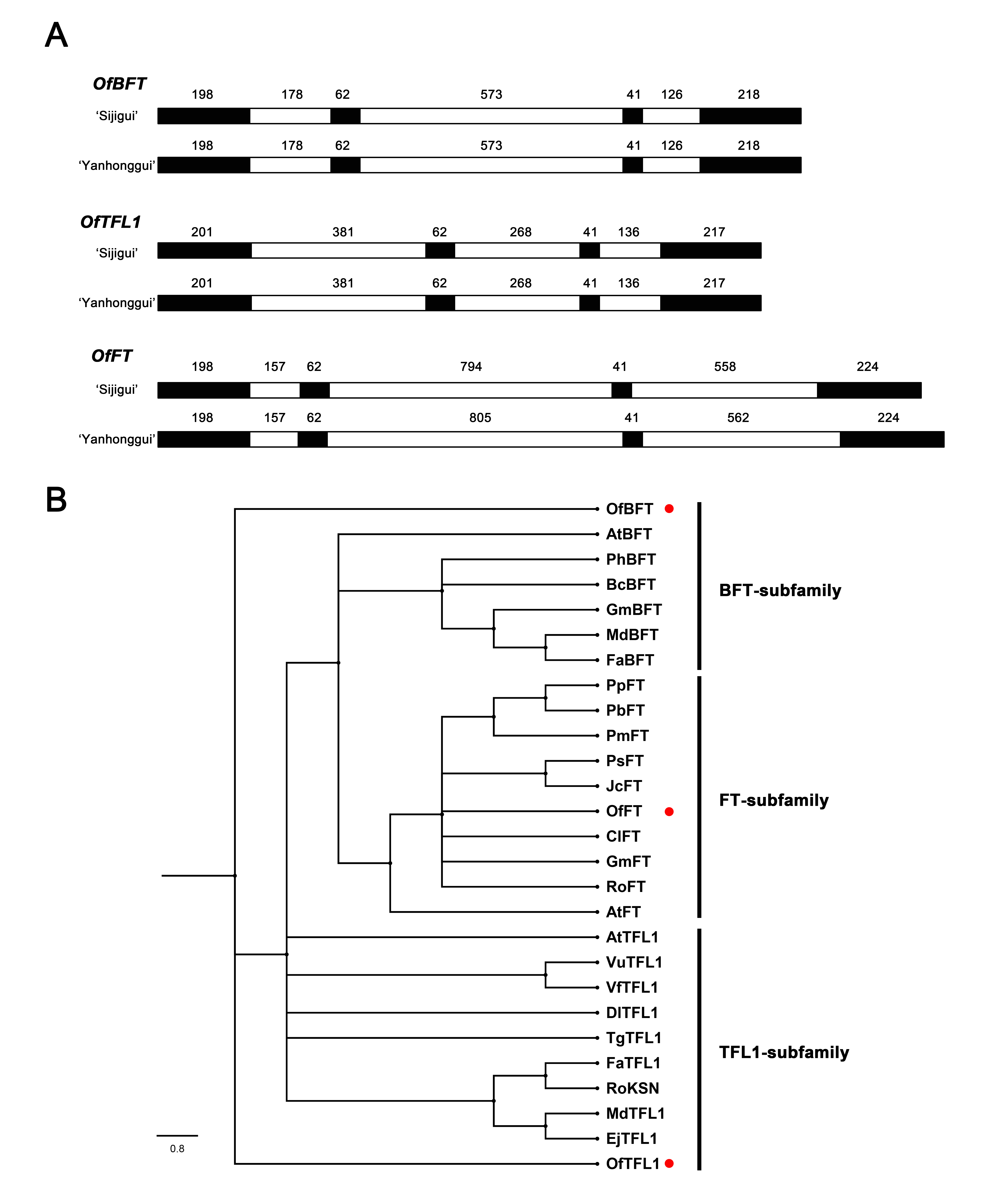

Supplement: Supplementary file 2 [file Image_2.tif]

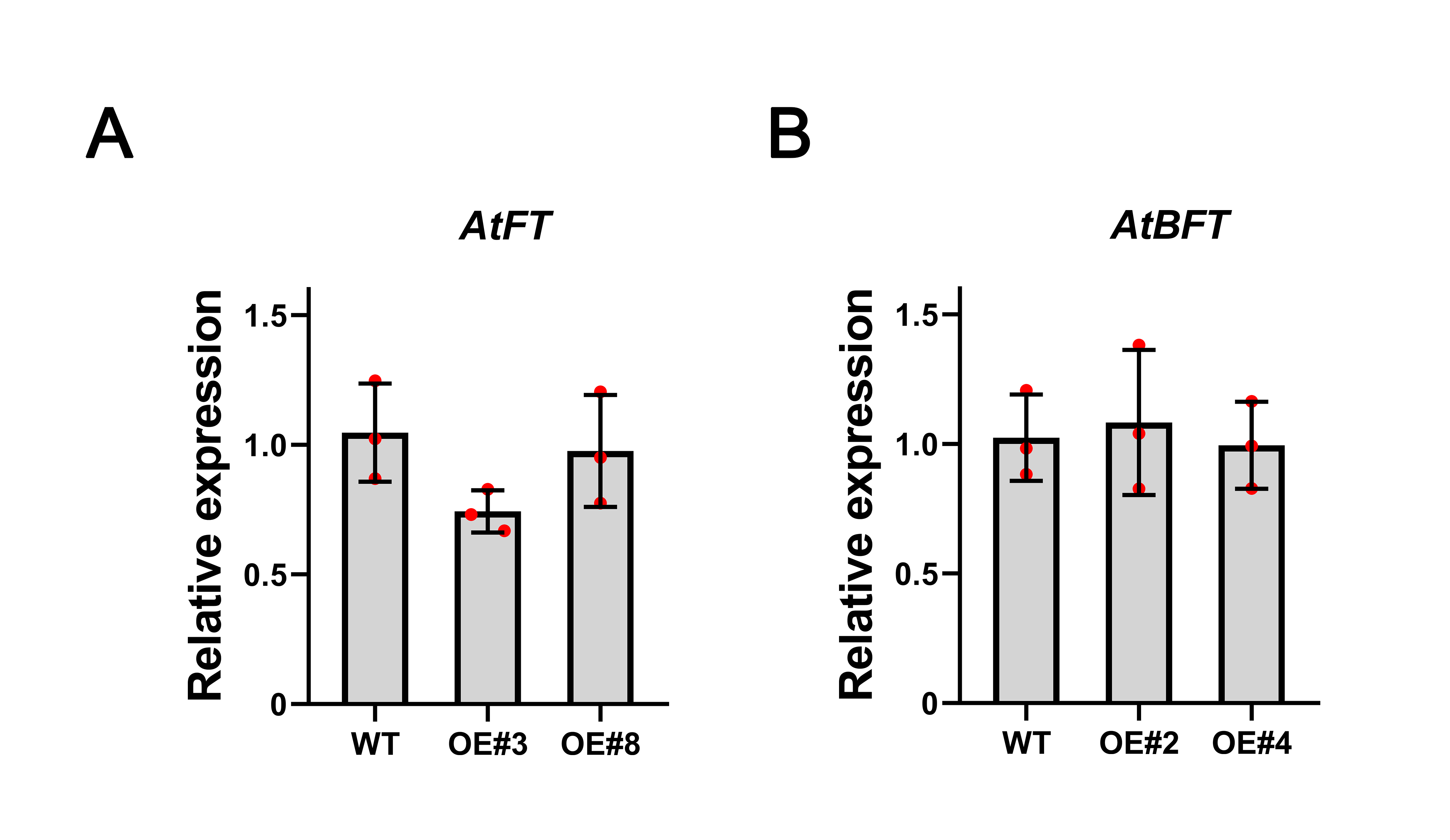

Supplement: Supplementary file 3 [file Image_3.tif]
