# Supplementary material for: Genetic studies on continuous flowering in woody plant Osmanthus fragrans
Source: Front Plant Sci. 2022 Nov 3;13:1049479. doi: 10.3389/fpls.2022.1049479 (PMC9671776; doi:10.3389/fpls.2022.1049479)
Supplement: Supplementary file 5 [file Table_2.docx]

Table S2 Summary of Illumina transcriptomic sequencing

| Sample | Raw reads number | Clean data size (bp) | Clean reads number | Clean data rate (%) |
| --- | --- | --- | --- | --- |
| WS-S0-1 | 21,948,388 | 6,536,468,838 | 21,918,257 | 99.86 |
| WS-S0-2 | 22,828,575 | 6,796,725,801 | 22,800,950 | 99.88 |
| WS-S0-3 | 24,243,411 | 7,222,851,708 | 24,206,473 | 99.85 |
| WS-S1-1 | 24,610,977 | 7,330,851,992 | 24,578,375 | 99.87 |
| WS-S1-2 | 29,543,103 | 8,805,778,988 | 29,506,994 | 99.88 |
| WS-S1-3 | 20,902,271 | 6,224,531,018 | 20,878,767 | 99.89 |
| AS-S0-1 | 21,726,270 | 6,472,596,342 | 21,702,064 | 99.89 |
| AS-S0-2 | 26,409,960 | 7,851,121,548 | 26,378,379 | 99.88 |
| AS-S0-3 | 27,312,642 | 8,124,117,766 | 27,276,775 | 99.87 |
| AS-S1-1 | 25,795,911 | 7,681,405,556 | 25,768,784 | 99.89 |
| AS-S1-2 | 32,246,921 | 9,599,740,754 | 32,208,845 | 99.88 |
| AS-S1-3 | 30,899,726 | 9,206,957,081 | 30,860,069 | 99.87 |
| AY-S0-1 | 26,935,077 | 8,005,729,384 | 26,909,881 | 99.91 |
| AY-S0-2 | 26,718,071 | 7,963,684,218 | 26,684,668 | 99.87 |
| AY-S0-3 | 31,550,353 | 9,390,316,424 | 31,515,149 | 99.89 |
| AY-S1-1 | 28,065,887 | 8,359,238,543 | 28,036,732 | 99.9 |
| AY-S1-2 | 30,549,268 | 9,096,945,429 | 30,516,793 | 99.89 |
| AY-S1-3 | 31,688,841 | 9,435,749,931 | 31,654,362 | 99.89 |

Note: WS: buds blooming in winter from ‘Sijigui’; AS: buds blooming in autumn from ‘Sijigui’; AY: buds blooming in autumn from ‘Yanhonggui’. S0: undifferentiation buds; S1: differentiation buds (mixed of inflorescence primordium differentiation stage and small floret primordium differentiation stage).
